# Potato Protein Fining of Phenolic Compounds in Red Wine: A Study of the Kinetics and the Impact of Wine Matrix Components and Physical Factors

**DOI:** 10.3390/molecules24244578

**Published:** 2019-12-13

**Authors:** Wenyu Kang, Richard A. Muhlack, Keren A. Bindon, Paul A. Smith, Jun Niimi, Susan E.P. Bastian

**Affiliations:** 1 School of Agriculture, Food & Wine, Waite Campus, The University of Adelaide, PMB 1, Glen Osmond, SA 5064, Australia; a1642412@adelaide.edu.au (W.K.); richard.muhlack@adelaide.edu.au (R.A.M.); jun.niimi@adelaide.edu.au (J.N.); 2 The Australian Wine Research Institute, Hartley Grove, Urrbrae, Adelaide, SA 5064, Australia; keren.bindon@awri.com.au; 3 Wine Australia, Industry House, Corner Hackney and Botanic Roads, Adelaide, SA 5000, Australia; Paul.Smith@wineaustralia.com

**Keywords:** wine, fining, potato proteins, gelatin, phenolics, tannin, Cabernet Sauvignon, design of experiments, factorial design, process optimisation

## Abstract

Producing wines within an acceptable range of astringency is important for quality and consumer acceptance. Astringency can be modified by fining during the winemaking process and the use of vegetable proteins (especially potato proteins) as fining agents has gained increasing interest due to consumers’ requirements. The research presented was the first to investigate the effect of a potato protein dose on the kinetics of tannin and phenolic removal compared to gelatin for two unfined Cabernet Sauvignon wines. To further understand the results, the influence of the wine matrix and fining parameters (including pH, ethanol concentration, sugar concentration, temperature, and agitation) were tested according to a fractional 2^5-1^ factorial design on one of the Cabernet Sauvignon wines using potato proteins. The results from the factorial design indicate that potato protein fining was significantly influenced by wine pH, ethanol concentration, fining temperature as well as an interaction (pH × ethanol) but not by sugar content or agitation. Insights into the steps required for the optimisation of fining were gained from the study, revealing that potato protein fining efficiency could be increased by treating wines at higher temperatures (20 °C, rather than the conventional 10–15 °C), and at both a lower pH and/or alcohol concentration.

## 1. Introduction

Astringency (a drying and rough in-mouth sensation) is considered to be one of the most important factors driving wine quality [1] and winemaking techniques are frequently applied in order to modulate wine astringency [2]. Too much astringency may render the wine difficult to drink, whilst too little may make the wine insipid and lower in complexity [1]. One way to manipulate astringency in wine is to alter the maceration process, ultimately affecting the extent or rate of transfer of phenolic compounds from the cap to the must/wine. This could involve pre-ferment cold maceration, which can potentially extract sufficient colour and flavours into wine but minimize the extraction of larger tannins [3,4,5]. Alternatively, extended maceration can increase tannin extraction into wine [2] and enhance astringency and possibly body. Other methods used to control astringency are for winemakers to make various additions to the ferments or finished wines. When astringency is lacking, winemakers may add oenological tannins (e.g., grape seed extract) to increase astringency perception. Conversely, if wine astringency is unacceptably high, astringency can be decreased or ‘softened’ by processes such as ageing or micro-oxygenation [6]. However, the ageing process can take long periods of time and despite being faster, micro-oxygenation is high in capital investment. Thus, a widely utilised convenient method to modify astringency is by a process known as fining. Fining involves an addition of agents in order to bind and remove phenolic components in wine in a targeted way, which, in turn, reduces astringency [7] and in doing so, possibly modifies the astringent sub-quality (nuanced differences in astringency texture perception) as well.

Traditionally, and to this day, winemakers use animal-based (i.e., gelatin, egg albumen, isinglass, and casein) and/or synthetic (i.e., polyvinylpolypyrrolidone, PVPP) products as fining agents to remove astringent compounds such as tannin in wine [7]. Nevertheless, using alternatives such as vegetable proteins has gained increased interest because of consumer demands due to the allergenic nature of animal-derived additives, or for ethical reasons [8]. One of the alternative vegetable-based fining products available on the market are potato proteins. Potatoes contain an active protein, patatin, which accounts for 40% of the total soluble potato protein and it is recovered from an aqueous by-product of potatoes [9]. Patatin ranges in molecular weight from 15 kDa to 120 kDa, with the majority around 40 kDa [10]. The patatin protein has a pI of 4.6, low solubility at wine pH [11] and has been demonstrated as a low risk for over-fining [12]. As one of the alternative agents, potato proteins have been shown to have a good capacity to fine wine phenolics and reduce grape must turbidity [12,13,14,15,16]. The fining efficiency of potato proteins was demonstrated to be similar to gelatin for phenolic removal and reduction of astringency sensation in commercial and model wine with added grape seed extract, but more effective than other traditional (casein, egg white, PVPP) and plant derived (pea, soy bean and rice) fining agents [12,16]. Overall flavour intensity and bitterness were not found to have been significantly affected by potato protein fining, but they can influence wine colour intensity and hue [16].

Currently, the fining efficiency of potato proteins (on phenols and turbidity) and the mechanism behind the interaction between potato proteins and components in wines are the focus of research in this field. Yet specifically, the time-dependent kinetics of fining with potato proteins have not yet been fully eludicated for red wines. In addition, wine matrixes vary greatly, but current knowledge on the use of potato proteins as fining agents for wine astringency modification has been limited to a small number of studies for a narrow range of red wine styles (Aglianico, Pinot Noir and Blaufränkisch) as well as a Cabernet Sauvignon unfined model wine [12,15,16]. Notably, the chemical environment of wine is very important for fining such as wine pH, polyphenol composition, and temperature [17]. These factors may influence the fining efficiency of potato proteins, either independently or cooperatively, and should therefore be considered for their potential influence on the efficacy of potato protein fining.

The intrinsic and extrinsic factors of wine can be investigated by the use of Design of Experiments (DoE). DoE (e.g., screening design, response surface design, and robust parameter designs, etc.) are very useful tools to examine complex processes because they allow the determination of the direct effects of each parameter and their interactions in a relatively small number of experiments [18]. Several studies to optimise vinification protocols using DoE techniques have been conducted. For instance, the optimization of using ultrasound to extract aroma compounds in white wine [19]; the sorption of wine volatile phenols by yeast lees [20]; the extraction of flavanols, phenolic acid and anthocyanin from Champagne grape varieties [21]; the control of haze-forming wine proteins by bentonite fining [22]; and anthocyanin transfer in simulated red wine fermentation [23] were all investigated by DoE. Thus, DoE can be a robust way to investigate the interactive processes which might occur in response to a technological intervention, in this case, the reduction of astringent compounds in wine by potato proteins.

The objectives of this research were firstly to investigate the kinetics of tannin and phenolic removal by fining using potato proteins at different doses on two real unfined Cabernet Sauvignon wines; and secondly, to investigate the interactive effects of key wine matrix variables on the fining of wine phenolics by potato protein using DoE.

## 2. Results and Discussion

### 2.1. Fining Kinetics of Potato Proteins Compared with Gelatin

**Figure 1:**
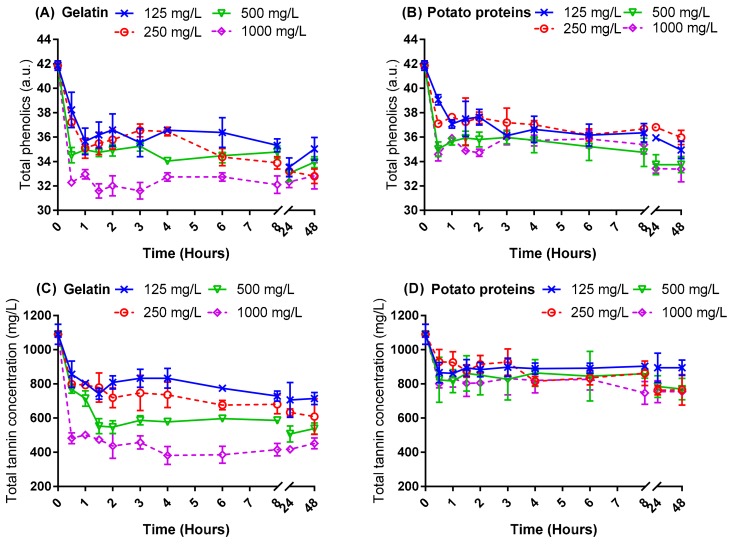
The fining kinetics of potato proteins compared with gelatin on unfined wine 1. (**A**,**B**) Total phenolics (absorbance units), and (**C**,**D**) total tannin (mg/L, epicatechin eq.).

**Figure 2:**
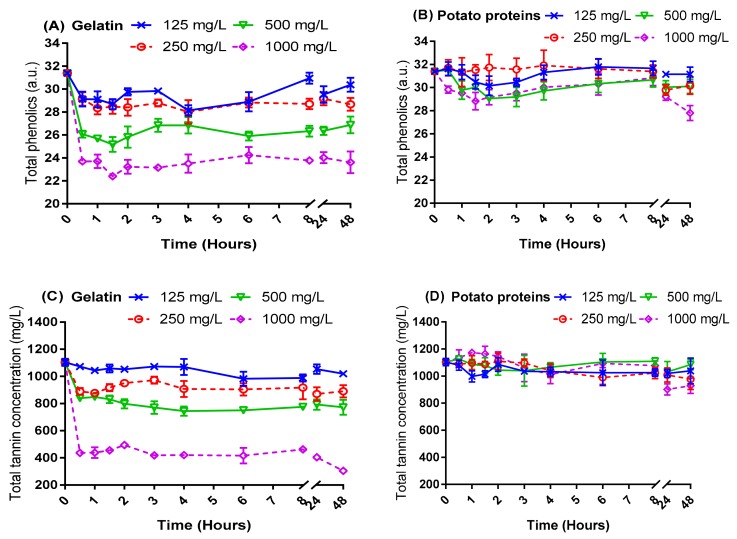
The fining kinetics of potato proteins compared with gelatin on unfined wine 2. (**A**,**B**) Total phenolics (absorbance units), and (**C**,**D**) total tannin (mg/L, epicatechin eq.).

Fining experiments were performed on two unfined red wines to study the changes in total phenolics and total tannin concentrations after the addition of potato proteins and gelatin (Figure 1 and Figure 2). The data were analysed by repeated measures analysis of variance (RMANOVA) with the Huynh-Feldt correction applied (Table 1), as the values of ε were greater than 0.75 from Mauchly’s sphericity test [24].

As shown in Figure 1 and Figure 2 as well as Table 1, the concentration of total phenolics and tannin generally decreased as the dose of gelatin was increased. With the exception of total phenolics in wine 1, a concentration-dependent trend for phenolics reduction using potato protein was not as strong as that observed for gelatin. At the same dose of fining agent applied, gelatin consistently brought about a greater reduction in total phenolics and tannin than potato proteins, for both wines studied. This finding was consistent with previous observations [16] which investigated a Cabernet Sauvignon model, unfined wine. However, the fining response differed between the two wines for both protein types applied. For instance, the addition of 125 mg/L gelatin significantly reduced tannin concentration in wine 1, but to achieve a similar fining response, a 500 mg/L dose was required for wine 2. Similarly, total phenolics were significantly reduced by potato proteins at a dose of only 125 mg/L in wine 1 but required a dose equal to or greater than 500 mg/L in wine 2 to bring about a statistically significant fining effect.

In addition, one hour of fining time was sufficient for both fining agents to achieve maximal adsorption of phenolics and tannin in wine 1. Furthermore, for gelatin, and less obviously for potato, an increased fining dose (i.e., 500 and 1000 mg/L) resulted in a maximum reduction of phenolics and tannins in a reduced time of 30 min. Given typical wine industry scale logistics, this bodes well for the use of in-line dosing of fining agents to remove phenols as opposed to batch fining and racking off fining lees.

Based on this kinetic study, it was discovered that wine conditions were important for fining, no matter which type of fining agent was used. As the fining efficacy observed in wine 1 was better than wine 2, hypotheses were made that the different fining efficacies were caused by the different phenolic profiles between the two wines and/or differences in basic chemical parameters (such as acid, sugar, and alcohol). Thus, the impact factors for potato proteins fining were further resolved in the current work.

### 2.2. Relevance of the Wine Phenolic Profile in the Response to Fining Agent Addition

As shown in Figure 1 and Figure 2, wine 1 had a higher concentration of total phenolics than wine 2, at 41.86 and 31.39 absorbance units (a.u.), respectively, but had a similar initial tannin concentration. Based on this observation, it was considered that differences in phenolics other than tannin (non-polymeric material) might account for the differences in the efficacy of potato protein fining between the two wines. Furthermore, given that tannin concentration was similar between wines 1 and 2, but very different responses were found for potato protein fining efficacy between the two wines, it was hypothesised that differences in tannin composition might be useful to explain these effects.

Therefore, tannins were isolated from the two wines and analysed (Table 2). Generally, it was observed that the tannins from each wine were compositionally similar in terms of subunit composition. An important difference between the two wines was found for tannin molecular mass (MM), measured both by phloroglucinolyisis and gel permeation chromatography (GPC). In terms of three-dimensional tannin size, the GPC measurement is considered to be more accurate, as it accounts for the hydrodynamic volume of the tannin material and gives an estimate of relative polydispersity [25]. According to the GPC result, tannin size in wine 2 was larger than wine 1. Theoretically, for proteins are observed to have a stronger binding capacity for larger than smaller tannins, when other structural attributes are similar, as was the case in the current study [26]. However, the fining efficacy for tannin was higher for wine 1 relative to wine 2, which did not support this hypothesis. This result suggests that the different effectiveness of fining by proteins observed for the two wines was not primarily due to differences in tannin composition or size but was more likely to be due to the influence of other chemical parameters within the wine matrix.

It was therefore considered that while, in general, the phenolic profiles of wine affect the final outcome of a fining treatment, other wine compositional parameters should not be ignored. Hence, the impact of other basic factors (such as pH, ethanol concentration, and sugar concentration) which can differ within different wine matrices, were further investigated in this study for their potential impact on the efficacy of fining by potato protein.

### 2.3. Experimental Design for Potato Proteins Fining

A 2^5-1^ fractional factorial experimental (one-half screening) design was used to determine the influence of basic wine and processing variables (during fining) on wine total phenolics and tannin concentration. Based on the results of the previously described kinetic study of fining by potato proteins where a reduction in polyphenols was observed more readily, wine 1 was selected for further experimentation via DoE. Furthermore, from the results of the kinetic study, 1 g/L and 48 h were chosen as the fining dose and contact time respectively, to ensure that a significant fining response would be observed.

**Figure 3:**
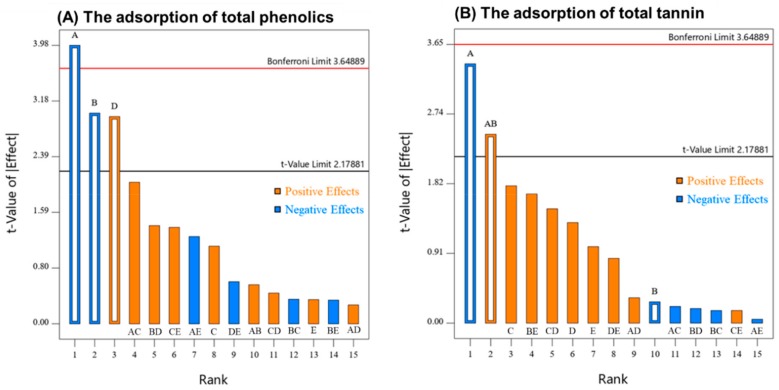
Pareto chart for the responses of (**A**) total phenolics adsorption and (**B**) total phenolics adsorption by potato proteins in the 2^5-1^ fractional factorial experimental. Factors A to E were pH, ethanol concentration, sugar concentration, temperature and agitation, respectively.

The effects (factors and their interactions) from the 2^5-1^ design on both responses were displayed in a Pareto chart (Figure 3). By running the ANOVA on the 2^5-1^ fractional factorial design, the factors of pH, ethanol, and temperature were found to be significant for total phenolics removal, however, only pH and an interaction (pH × ethanol) were significant for the tannin adsorption (all *p* values < 0.01). As sugar concentration and agitation were not significant for either response, the fractional factorial design was consolidated into a full factorial design (2^3^) of the remaining significant factors (with each remaining factor combination now consisting of six replicates), permitting the effect and significance of all two and three factor interactions between pH, temperature and ethanol concentration to be determined.

#### 2.3.1. Modelling the Adsorption of Total Phenolics by Potato Protein Fining

The ANOVA on the consolidated design to 2^3^ was determined, and the model F value was significant at 5.88 (*p* = 0.0001), while the “lack of fit” was not significant (F = 1.10, *p* = 0.3907). Meanwhile, the F values in this model for pH, ethanol concentration and temperature were 17.21, 9.85 and 9.52, respectively (all *p* values < 0.01). This confirmed the observation above that the factors pH, ethanol concentration and temperature were all relevant in determining the potato protein fining response for the removal of total wine phenolics, but their interaction (two-way and three-way) were not significantly important.

**Figure 4:**
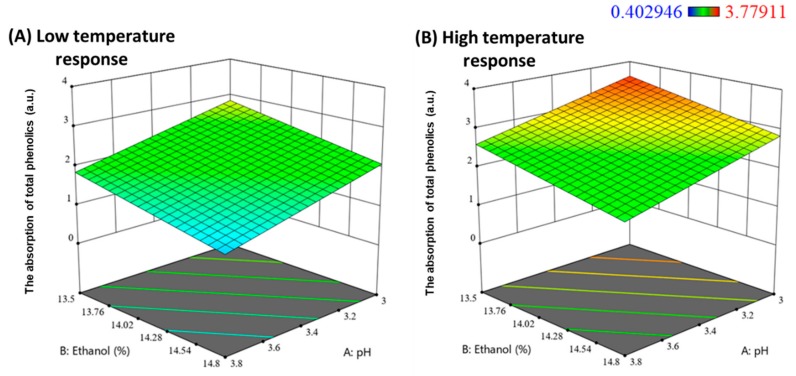
Response surface showing the adsorption of total phenolics as a function of pH and ethanol concentration for (**A**) ‘low’ and (**B**) ‘high’ temperature (10 °C and 20 °C respectively).

A three-dimensional response surface diagram for phenolics adsorption as a function of both pH and ethanol concentration at both low (Figure 4A) and high (Figure 4B) temperatures was visualised. Both pH and ethanol were found to exert negative effects, which indicates that higher pH and ethanol concentration diminished the efficacy for total phenolics removal by the specified potato protein dose. pH is known to have an influence on polyphenol-protein interactions [27], where pH can alter the ionic charge of proteins [28]. To interpret the current results, it is possible that an increased charge on the surface of the potato protein may have resulted at lower pH, which caused a stronger electrostatic interaction with polyphenols. Prior studies have demonstrated that there is generally a combination of both hydrophobic and hydrogen bonding between proteins and tannins, the degree of which depends on structure and matrix differences [29,30]. Ethanol concentration is also known to have an impact on the mechanism of interaction between wine phenols and poly(l-proline). A transition was found from a combination of hydrophobic and hydrogen bonding at 10% (*v*/*v*) ethanol, with a small increase to 15% (*v*/*v*) ethanol, resulting in interactions which were primarily via hydrogen bonding [31]. In light of these observations, to interpret the results of the present study, it may be hypothesised that since a higher ethanol concentration in wine was found to reduce phenolic adsorption and precipitation by potato proteins, that both hydrophobic and hydrogen bonding were important in driving the fining response.

Conversely, another significant main effect, fining temperature, was found to positively influence the adsorption of total phenolics by potato protein fining. There was approximately a 1 a.u. difference between the low- and high-fining temperature responses when other factors were kept constant. This observation caused by different temperatures was also consistent with other wine fining literature with other fining agents [22,32].

From a processing perspective, the results determined by the DoE suggested potato proteins fining efficiency (on total phenolics adsorption) would be optimised by treating wines at higher temperatures rather than at normal storage temperatures for red wines (10 °C to 15 °C) Although this study did not vary the amount of fining agent, an adjustment of wine pH and alcohol to a lower level could possibly reduce the amount of fining agent required. In turn, this would reduce fining agent costs, whilst lowering risks of over-fining other wine components such as colour and aroma. However, manipulating alcohol through earlier harvest, water addition or reverse osmosis, might lead to reduced wine quality in itself [33,34,35], so wine producers would need to consider what the best option may be. Overall, pH adjustment is likely the best parameter to manipulate in order to enhance potato fining efficacy.

#### 2.3.2. Modelling the Adsorption of Tannin by Potato Protein Fining

The tannin response model of the 2^3^ full design was analysed, the model F value in ANOVA was 5.20, which indicated that there was only a 1% chance that differences were due to noise, hence the significance of the model was confirmed. The “lack of fit” was also not significant (*F* = 0.04, *p* = 0.9861). Adsorption of total tannins after fining were significantly influenced by pH (*F* = 11.52, *p* = 0.005) likely due to its impact on protein-tannin interactions as discussed for phenolics in Section 2.3.1. Furthermore, the significant pH × ethanol interaction observed from the 2^5-1^ design analysis was confirmed in this 2^3^ design analysis (*F* = 6.11, *p* = 0.029).

**Figure 5:**
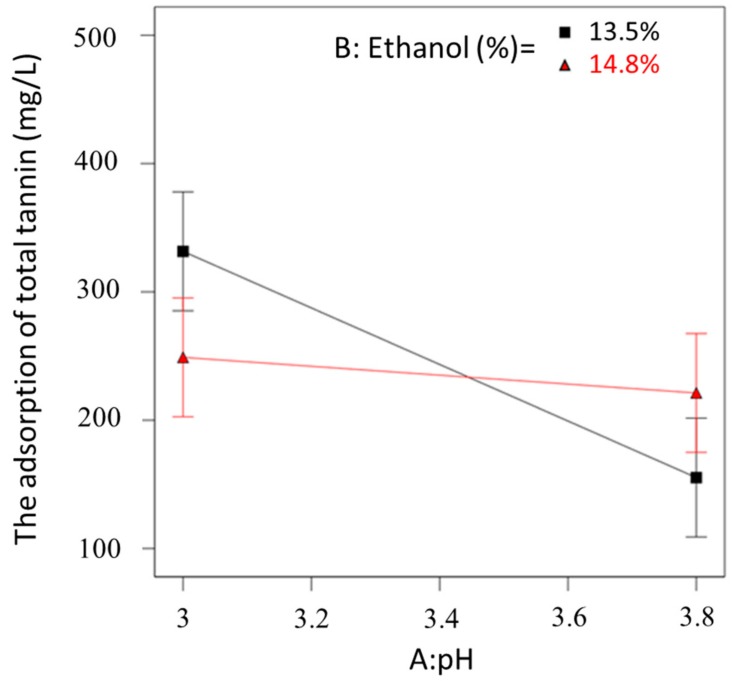
The interaction plot between the factors of pH and ethanol concentration on the adsorption of total tannin.

This interaction (pH × ethanol) positively influenced the removal of wine tannin, and it may be visualised in Figure 5. The fining efficacy was significantly decreased with increasing pH when the ethanol concentration was low. However, the pH change did not significantly influence the tannin removal efficacy when the wine ethanol was high. Insights of impact of various processing parameters on tannin removal were gained that treated wines with potato proteins at lower pH and ethanol concentration. However, there is no need for winemakers to modify wine pH in order to enhance fining efficiency, if the alcohol concentration is high.

Importantly, in the current study, sugar concentration in wine was not a significant factor for the fining treatment with potato proteins. Similarly, agitation during fining did not significantly impact on either the adsorption of total phenolics or tannin. Based on the observations from the current study, future work could be expanded to apply the DoE methodology on a greater range of red wines, with greater compositional variation. Furthermore, wine is a very complex matrix and consists of other components such as metal ions and polysaccharides [36,37], the impact from other components should be deciphered in the future.

## 3. Materials and Methods

### 3.1. Wine Samples and Fining Agents

Two Australian Cabernet Sauvignon (*Vitis vinifera*) wines from vintage 2019 were used as the unfined wines (Table 3). The fining experiments were conducted immediately after wine fermentation and cross flow filtration for wine 1 and fermentation followed by cold settling and racking for wine 2.

The potato protein was purchased from Laffort Australia (VEGECOLL^®^, Lot 117. Woodville North, SA, Australia). In addition, a powdered gelatin that is conventionally used in the wine industry, was used as a reference standard fining agent for comparison (Lot 161129, purchased from Laffort Australia). Before the fining process was initiated, each agent was solubilized in Milli-Q water as a stock solution (50 g/L, stirred for 12 h at 20 °C), to ensure accurate additions [38].

### 3.2. Chemicals

Reagents and reference compounds (≥97% purity) used for the methyl cellulose precipitable (MCP) tannin assay, the modified Somers assay, and high-performance liquid chromatography (HPLC) were purchased from Sigma-Aldrich (Castle Hill, NSW, Australia).

Ethanol (96%), d-(+)-Glucose (≥99.5% purity), sodium hydroxide, and hydrochloric acid (37%) were purchased from Chem-supply (Gillman, SA, Australia), Sigma-Aldrich, Rowe Scientific (Lonsdale, SA, Australia), and Merck (Bayswater, VIC, Australia), respectively.

Milli-Q water (Millipore, North Ryde, NSW, Australia) was used for all solution preparations.

### 3.3. Fining Kinetics of Potato Proteins Compared with Gelatin

Fining experiments were performed on a 500 mL scale in both unfined wines (in 500 mL Schott bottles). The stock solutions of fining agents were serially diluted 1 in 2, to generate the following concentrations of 125, 250, 500 and 1000 mg/L (i.e., a total of eight treatments for each wine). After mixing (by Ratek OM11 orbital mixer, 150 rpm for 2 min at 20 °C), the headspace of each treated wine was filled with N_2_ gas to avoid oxidation. Thereafter, all fining treatments were settled in the dark at 20 °C. After 0.5, 1, 1.5, 2, 3, 4, 6, 8, 24 and 48 h of treatment with fining agents, 10 mL of each treated wine was sampled from the same position in the Schott bottle (midpoint of the bottle and 1.5 cm below the liquid surface). Aliquots were immediately transferred into 15 mL tubes and centrifuged at 4000 rpm for 5 min (by Eppendorf centrifuge 5810). The supernatants of each treated wine were recovered into new tubes. Total tannin concentration and total phenolics were measured for all samples. The unfined wine had an addition of the equivalent volume of Milli-Q water and was set as time point ‘0 h’ in this kinetic study (1.25 mL water in 500 mL unfined wine, the same amount as fining agent addition). All fining trials were conducted in triplicate.

### 3.4. Phenolics Analyses

Total phenolics for samples was measured by the modified Somers assay [39] in technical triplicate. Total tannin concentration was measured through the high throughput MCP tannin method [39] in a technical duplicate. The tannin concentrations were calculated as epicatechin equivalents (g/L) from an epicatechin standard curve (Figure S1).

In addition, tannins from the two unfined wine samples were isolated using solid phase extraction [40] and analysed by HPLC following phloroglucinolysis (Agilent 1100 (Melbourne, VIC, Australia)) [41] to determine the subunit composition, mean degree of polymerization (mDP), and molecular mass (MM (phloro)). The molecular mass of tannins was also determined by gel permeation chromatography (MM (GPC)) [25] on an Agilent 1200. The tannin isolation and following measurements were performed in triplicate per sample.

### 3.5. Experimental Design for Potato Proteins Fining

A 2^5-1^ fractional factorial experimental design (one-half screening design) was used to determine the influence of the 5 variables (pH, ethanol concentration, sugar concentration, temperature, and agitation) at two levels each on the ability of potato proteins to remove total phenolics and tannin in wine. The factors and their associated levels (typical levels found in table wines) are summarised in Table 4. One of the two wines (Wine 1) was selected for the study. The pH of the wine was altered by using a few drops of sodium hydroxide (50%) and hydrochloric acid (37%), respectively. The wine ethanol concentration was manipulated by using the same volume (1 mL addition in every 75 mL Wine 1) of Milli-Q water and 96% ethanol. Although the dilution effect in wine from the water and ethanol addition might not be the same, the assumption was made that it was as the same volume was added of each. Glucose was used to modify the sugar concentration of the wine. The corresponding temperatures were achieved using temperature-controlled rooms, and the agitation was achieved by a Ratek mixer (at 100 rpm).

The experiment was performed with the treatment combinations generated by the Design Expert software (version 11, Minneapolis, MN, USA) shown in Table 5. The fining process in DoE was conducted on a 50 mL scale, but all other experimental details (such as the process of fining agent addition and centrifugation to remove fining agent) were kept the same as the kinetic study. A fining agent dose of 1000 mg/L and 48 h were chosen as the fining concentration and time for the DoE experiment, to ensure that a significant fining response would be observed. The adsorption of total phenolics and total tannin in wine was measured as two responses in the factorial design.

The experiments were performed in triplicate.

### 3.6. Data Analyses

The data of the fining kinetic study (including the ‘0 h’ time point) were analysed by RMANOVA in SPSS statistics (ver. 26; IBM Corporation, Chicago, IL, USA). RMANOVA was conducted independently on the total phenolics and tannin measures from the two unfined wines. The Huynh–Feldt correction was applied due to the violation of the sphericity assumption of RMANOVA which was determined by Mauchly’s test. The adsorption of total phenolics and tannin was obtained by subtracting the values of total phenolics and tannin before and after fining, and the data for the factorial design were processed via the Design Expert software (multiple linear regression).

## 4. Conclusions

This study was the first to investigate the fining kinetics of potato proteins on phenolic components in Cabernet Sauvignon wines. The fining performances were driven by different conditions in wines, including the phenolic profiles and basic chemical composition of the matrices. This work was also the first to resolve the main impact factors for reducing wine astringent compounds (total phenolics and total tannin) by using potato proteins. Potato protein fining was significantly influenced by wine pH, ethanol concentration, fining temperature as well as the pH × ethanol interaction, but not by sugar content or agitation. Winemaking optimisation insight was gained by the factorial design experiment in this work, in that reduction in the amount of potato protein required may be achieved by fining wines either at higher temperatures (rather than normal storage temperatures), at a lower pH, and/or lower alcohol concentration.

## Figures and Tables

**Table 1:** The results of the repeated measures ANOVA with the Huynh-Feldt correction.

			Concentration (mg/L)			
			125	250	500	1000
Wine 1	Gelatin	Total phenolics	*	***	***	***
		Total tannin	**	**	***	***
	Potato proteins	Total phenolics	***	*	***	***
		Total tannin	ns ^a^	ns	ns	*
Wine 2	Gelatin	Total phenolics	**	**	***	***
		Total tannin	ns	ns	*	***
	Potato proteins	Total phenolics	ns	ns	*	**
		Total tannin	ns	ns	ns	*

^a^ ns: no significant difference. Symbols *, ** and *** denoted for *p* value < 0.05, 0.01 and 0.001 respectively, showing a significant change was detected across the fining period (eleven time points across 48 h).

**Table 2:** The tannin composition (mean ± standard deviation) of the two unfined wines in the current study.

	mDP ^a^	Epigallocatechin (%) ^a^	Epicatechin Gallate (%) ^a^	Mass Conversion (%) of Phloroglucinolysis ^a^	MM (phloro) (g/mol) ^a^	MM (GPC) (g/mol) ^b^
Wine 1	8.32 ± 0.06	38.7 ± 0.0	2.3 ± 0.0	45.5 ± 0.6	2495 ± 19	1628 ± 0
Wine 2	8.76 ± 0.25	36.4 ± 0.0	2.8 ± 0.0	44.0 ± 0.9	2631 ± 77	1935 ± 3

^a^ Determined by phloroglucinolysis. ^b^ Determined by gel permeation chromatography at 50% elution.

**Table 3:** The oenological parameters of the unfined wines used in the current study.

	Wine 1	Wine 2
Grape Source	Limestone Coast, Australia	McLaren Vale, Australia
Yeast strain	Maurivin AWRI 796	Enartis Ferm red fruit
Malo-lactic fermentation Strain	CHR Hansen CH16	LALLEMAND VP41
Oak influence	No	No
pH ^a^	3.51	3.77
Tartaric acidity (g/L) ^a^	6.67	5.90
Malic acid (g/L) ^a^	<0.40	<0.40
Volatile acidity (g/L) ^a^	0.44	0.76
Alcohol (%) ^a^	13.73	15.60
Residual sugar (g/L) ^a^	0.17	3.30
Free sulfur dioxide (mg/L) ^b^	29	30
Total sulfur dioxide (mg/L) ^b^	48	59

^a^ Wines were measured by the Australian Wine Research Institute’s (AWRI) Commercial Services Laboratory by Winescan method. ^b^ Wines were measured by the Australian Wine Research Institute’s (AWRI) Commercial Services Laboratory by the method of sulfur dioxide free and total (Gallery).

**Table 4:** Experimental factors for the two-level fractional factorial experimental (one-half).

Factor	Description	Low Level	High Level
A	pH ^a^	3.00	3.80
B	Ethanol (%) ^b^	13.5	14.8
C	Sugar (g/L) ^c^	0.16	8.00
D	Temperature (°C)	10	20
E	Agitation	No	Yes

^a^ Wine samples were measured for pH by using a Mettler Toledo T50 Autotitrator (Port Melbourne, VIC, Australia). ^b^ The ethanol concentration was determined with the Anton Paar Alcolyzer Wine ME and DMA 4500M (North Ryde, NSW, Australia). ^c^ The sugar content were determined via Chemwell^®^ 2910 Automated EIA and Chemistry Analyser (Awareness Technology, Palm City, FL, USA) with the Megazyme K-FRUGL test kits (Chicago, Illinois, USA).

**Table 5:** Treatment combinations for the fractional factorial experimental (one-half) design. Factors A to E were pH, ethanol concentration, sugar concentration, temperature and agitation, respectively.

	Treatments *		Treatments
1	AbcDE	9	abCDE
2	AbCDe	10	ABcDe
3	ABCDE	11	aBCDe
4	Abcde	12	aBcDE
5	aBCdE	13	AbCdE
6	aBcde	14	ABCde
7	abCde	15	abcdE
8	ABcdE	16	abcDe

* A high level of any factor in the treatment combination is denoted by the capital letter and a low level of a factor is denoted by lowercase letter.

## Supplementary Materials

The following are available online, Figure S1: The standard curve of tannin concentration (epicatechin eq.) of methyl cellulose precipitable method.
