# Supplementary material for: Potato Protein Fining of Phenolic Compounds in Red Wine: A Study of the Kinetics and the Impact of Wine Matrix Components and Physical Factors
Source: Molecules. 2019 Dec 13;24(24):4578. doi: 10.3390/molecules24244578 (PMC6943501; doi:10.3390/molecules24244578)
Supplement: Supplementary file 1 [file molecules-24-04578-s001.pdf]

Supplementary Materials:

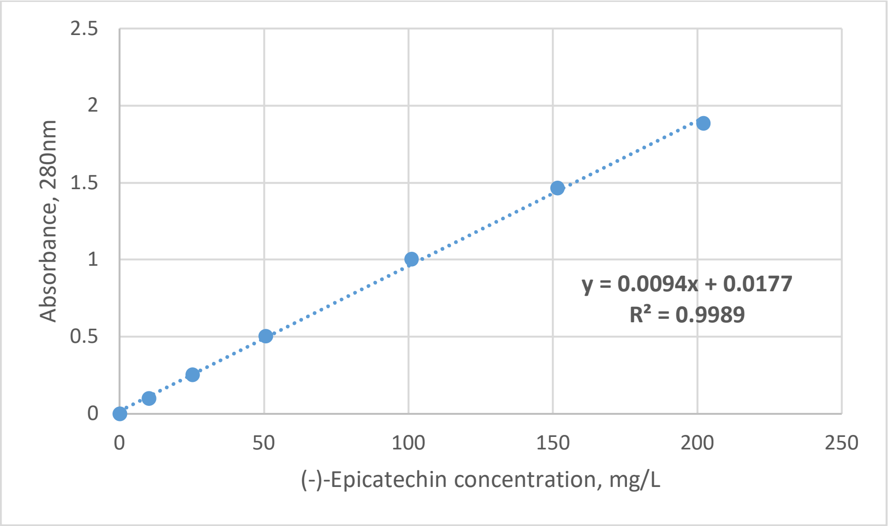

Figure S1. The standard curve of tannin concentration (epicatechin eq.) of methyl cellulose precipitable method.
